# Structural insights into Resveratrol’s antagonist and partial agonist actions on estrogen receptor alpha

**DOI:** 10.1186/1472-6807-13-27

**Published:** 2013-10-25

**Authors:** Sandipan Chakraborty, Anait S Levenson, Pradip K Biswas

**Affiliations:** 1 Laboratory of Computational Biophysics & Bioengineering, Department of Physics, Tougaloo College, 500 West County Line Road, Toogaloo, MS 39174, USA; 2 Saroj Mohan Institute of Technology, Hooghly, West Bengal 712512, India; 3 Cancer Institute and Department of Pathology, University of Mississippi Medical Center, Jackson, MS 39202, USA

**Keywords:** Resveratrol, Estrogen Receptor, Agonist, Antagonist, Phytoestrogen, Helix 12, Molecular dynamics simulation, Hydrogen bonding

## Abstract

**Background:**

Resveratrol, a naturally occurring stilbene, has been categorized as a phytoestrogen due to its ability to compete with natural estrogens for binding to estrogen receptor alpha (ERα) and modulate the biological responses exerted by the receptor. Biological effects of resveratrol (RES) on estrogen receptor alpha (ERα) remain highly controversial, since both estrogenic and anti-estrogenic properties were observed.

**Results:**

Here, we provide insight into the structural basis of the agonist/antagonist effects of RES on ERα ligand binding domain (LBD). Using atomistic simulation, we found that RES bound ERα monomer in antagonist conformation, where Helix 12 moves away from the ligand pocket and orients into the co-activator binding groove of LBD, is more stable than RES bound ERα in agonist conformation, where Helix 12 lays over the ligand binding pocket. Upon dimerization, the agonistic conformation of RES-ERα dimer becomes more stable compared to the corresponding monomer but still remains less stable compared to the corresponding dimer in antagonist conformation. Interestingly, while the binding pocket and the binding contacts of RES to ERα are similar to those of pure agonist diethylstilbestrol (DES), the binding energy is much less and the hydrogen bonding contacts also differ providing clues for the partial agonistic character of RES on ERα.

**Conclusions:**

Our Molecular Dynamics simulation of RES-ERα structures with agonist and antagonist orientations of Helix 12 suggests RES action is more similar to Selective Estrogen Receptor Modulator (SERM) opening up the importance of cellular environment and active roles of co-regulator proteins in a given system. Our study reveals that potential co-activators must compete with the Helix 12 and displace it away from the activator binding groove to enhance the agonistic activity.

## Background

Resveratrol (RES) (3,5,4′-trihydroxy-*trans*-stilbene) is a naturally occurring stilbene commonly found in grapes and red wine [1]. Resveratrol has various health benefits, such as cardiovascular, neuroprotective, anti-oxidant, anti-inflammatory, anti-diabetic, anti-viral, and cancer preventive properties [2-7]. Resveratrol has been characterized as a phytoestrogen based on its ability to compete with 17β-estradiol (E2) for binding to and modulating the activity of estrogen receptor alpha (ERα) [8]. However the biological effects of RES on ERα are still highly controversial. Experimental studies performed using different cell lines and reporter constructs suggest that the estrogenicity of RES, both in terms of the potency and degree of agonism depends on the cell type, the specific sequence and promoter context of the estrogen response elements (EREs), gene of interest, ER isoforms, and the assays used [7]. Superestrogenic properties of RES have first been reported in MCF-7 human breast cancer cells transfected with reporter-gene constructs, [8] and then were confirmed in additional studies with MCF7 and HepG2 cells by others [9,10]. Full agonism of RES was observed in breast cancer cells expressing endogenous ERα [11] and stably transfected with wild type and mutant (D351Y) ERα [12,13]. However, in other cell types (COS-1, kidney; BG-1, ovarian; CHO-K1, ovarian) transfected with ERα, only partial agonism was observed depending on the ERE-reporter [8,14,15] while the agonistic stimulation of cell growth were observed in two other studies in non-breast cancer cells [16,17]. In addition, RES antagonism or “no agonism” was also reported in E2-treated MCF7 cells, mammary tumor models and in the reproductive and nonreproductive estrogen target tissues *in vivo*[18-22]. Altogether, RES appears to be a mixed agonist/antagonist, a land mark feature or characteristic of selective ER modulators (SERMs) [23-25]. The gene expression profiling of breast cancer cells transfected with ERα and treated with E2, RES, several SERMs and pure antiestrogen ICI revealed substantial overlap between RES- and SERM-induced gene modulations confirming postulated mixed agonist/antagonist character of RES [24,25].

The mixed agonist/antagonist nature of RES remains of great interest considering its potential to have an impact on human health. While as a natural SERM, the partial estrogenic activity of RES could provide health benefits in cardiovascular system, bone tissues, and Alzheimer’s disease, there is a concern for its adverse side effects if it is used as a preventive or therapeutic agent for hormone-dependent cancers, particularly in ERα positive breast cancers.

Resveratrol binds to ERα and is able to compete with classical estrogen E2 [8,14,15]. Binding of estrogenic ligand brings conformational changes suitable for the protein to dimerize, recruit co-activator proteins, bind to the ERE in the promoter region of target genes, and trigger gene transcription [26-28]. ERα exhibits discrete ligand-specific conformational changes: in the presence of SERM and pure antagonist, the AF2 helix, i.e., Helix 12 adopts a distinct conformation than the agonist bound ERα, preventing the co-activator binding [29-31]. Interestingly, in experiments with AF2 deleted mutant ERα, E2 and RES behaved differently in their ability to regulate TGFα expression suggesting possible conformational differences in ERα-E2 and ERα-RES complexes [13].

The structure of the RES-ER complex has not yet been experimentally determined. Molecular modeling suggested RES to form more hydrogen-bonding with ERα than DES and thus have the potential to generate different conformation of the protein [32,33]. However, the resulting receptor dynamics upon RES binding has never been explored. In the present study, we have investigated the conformational dynamics of the ERα ligand binding domain (LBD) monomer and dimer bound to RES using highly suitable method of Molecular Dynamics (MD) simulation and examined the effect of RES binding in both classical agonist and antagonist conformations. We refer the agonist and antagonist conformation of ERα in terms of Helix 12 orientations as observed from the different ligands bound to ERα LBD crystal structures [28-30]. Our results provide for the first time the structural reasons for antagonist and partial agonist activity of RES exerted on ERα.

## Results and discussions

**Scheme 1:**
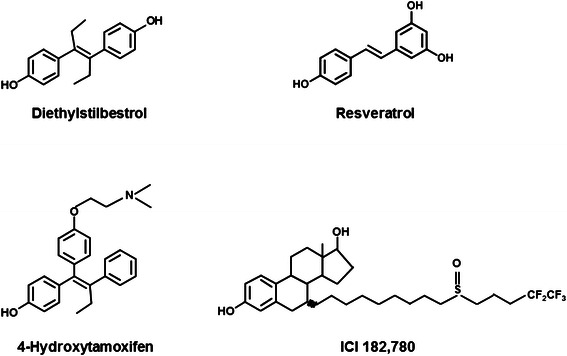
Chemical structures of four ERα ligands used in this study: Diethylstilbestrol, Resveratrol, 4-Hydroxytamoxifen, ICI 182,780.

We used MD simulation to systematically study the effect of RES binding to ERα LBD in monomer and homo-dimer structures. We refer to agonist or antagonist structure in terms of the relative orientation of helix-12 position as has been observed in DES and 4-hydroxy tamoxifen (4-OHT) bound crystal structures for ERα LBD [30]. When estrogenic DES is bound to ERα LBD, helix-12 lays over the ligand binding pocket. However, when SERM 4-OHT is bound to ERα LBD, helix-12 orients away from the ligand binding pocket and lays over the co-activator binding groove formed by residues from helices3, 4, and 5 and the turn connecting helices 3 and 4. Schematic representations of all the four ligands (Diethylstilbestrol, Resveratrol, 4-Hydroxytamoxifen and ICI 182,780) have been shown in Scheme 1.

### Effect of resveratrol binding on ERα monomers

**Figure 1:**
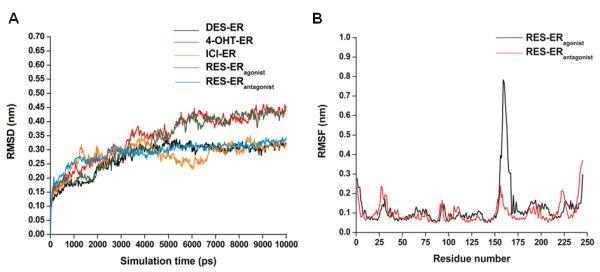
**Variation of dynamic parameters of ERα bound to different ligands obtained from MD stimulation. (A)** Variations in C_α_-RMSD of ERα monomer with simulation time. RES-ER_antgonist_, DES-ER and ICI-ER have highly stable complexes during simulation (*cyan, black and orange*), RES-ER_agonist_ and 4OHT-ER show high fluctuations (*green, red*). **(B)** RMSF profile of RES-ERα monomer. *Black* and *red* lines represent RES bound ERα in agonist and antagonist conformation, respectively. The peak in fluctuation corresponds to Helices 8 & 9 (residues 150 to 166).

Molecular dynamics simulations have been performed on RES bound to both agonist and antagonist ERα LBD monomers to gain insights into structural stability and conformational dynamics of the protein-ligand complexes and their dependency on the bound ligand subtypes (Figure 1).

The RMSD (root means square deviations) of the backbone C_α_ atoms of the simulated protein over time can be used to analyze the structural stability of the system. To understand the stability of RES bound ERα-LBD complexes and to compare them with ERα-LBD bound to known agonist, antagonist, and SERM ligands, we have performed molecular dynamics of RES bound with ERα-LBD agonist and antagonist monomers, DES bound with ERα-LBD agonist monomer, and 4-OHT and ICI bound with ERα-LBD antagonist monomer. As evident from Figure 1A, during the first 2 ns of the simulation, all the systems undergo conformational readjustments according to the bound ligand in the initial ERα structure and monotonically tend to reach an equilibrium state. When pure agonist (DES) or antagonist (ICI) is bound to the LBD, the complex reaches a stable equilibrium state during simulation. Resveratrol bound ERα-LBD antagonist conformation also reaches a stable equilibrium state during the simulation and its conformational dynamics is comparable with the pure agonist DES or pure antagonist ICI bound LBD. On the contrary, RES bound agonist ERα-LBD shows high fluctuations over its assumed equilibrium state during the simulation which is comparable to the SERM (4-OHT) induced dynamics of ERα-LBD.

To understand the structural basis for the observed differences in RMSD fluctuations between the RES bound agonist and antagonist ERα-LBD conformations during simulation, we have analysed the RMSF (root mean square fluctuations) per residues to identify the regions of high fluctuations. Results are summarized in Figure 1B. In general, the residue fluctuations for RES bound antagonist ERα are very comparable to the RES bound agonist ERα complex. We found that the observed high RMSD fluctuations of the RES bound ERα agonist complex are mainly due to the long loop region between Helix 8 and Helix 9 and N-terminal region of Helix 9 (from residue 150 to 166). This region is found to be highly flexible during the MD simulation for the RES bound agonist ERα. In contrast, Helix 12 has been found to be flexible in both RES bound ERα antagonist and agonist conformations.

**Figure 2:**
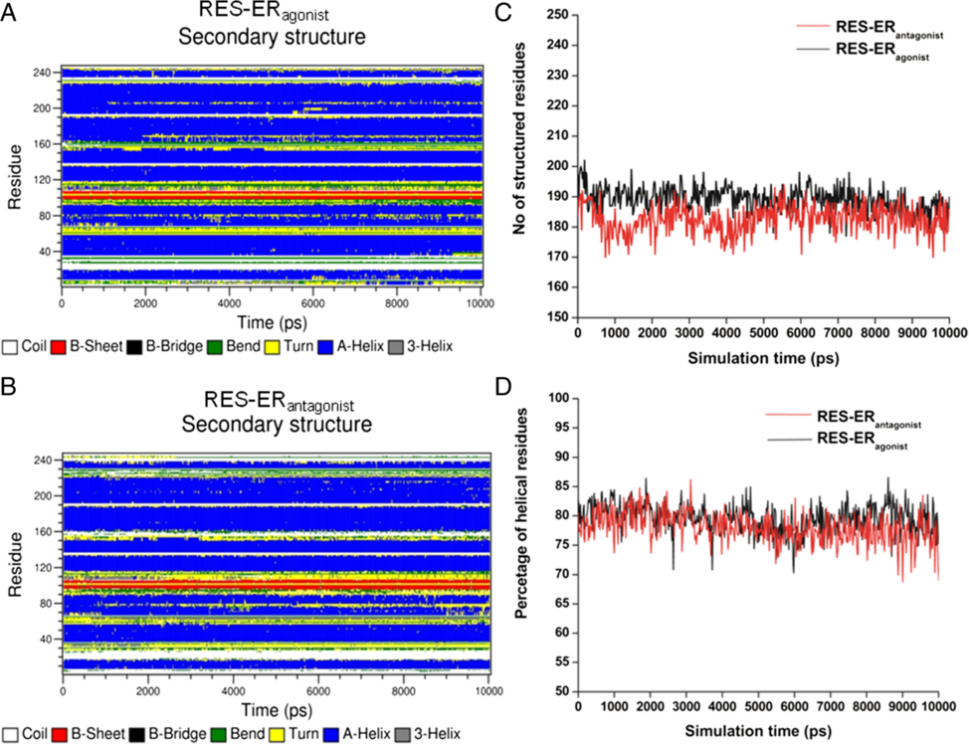
**Secondary structure profiles with simulation time. (A)** RES-ERαagonist complex; **(B)** RES-ERαantagonist complex. **(C)** Variation of structural content of ERα bound with RES during simulation. **D**: Variations of percentages of helical structures in RES-ERα complex with simulation time.

We then analyzed the effect of RES binding on the secondary structure profile of ERα agonist and antagonist conformations (Figure 2). ERα essentially constituted of 12 helices and one β-strand connected by short loop regions. Comparisons of secondary structure evolution of RES bound agonist (Figure 2A) and antagonist (Figure 2B) ERα reveal that the secondary structures of both the complexes are stable during the MD simulation. As evident from Figure 2C & D, for initial agonist conformation, there are 197 residues adopting a defined secondary structure and, during simulation, on average 189 ± 4 residues maintain their initial secondary structure. In case of RES-ERα antagonist complex, the initial structure has 184 residues with defined secondary structure and 182 ± 4 residues maintain their secondary structure during simulation. Thus, RES-ERα antagonist complex maintains its native secondary structure better during the simulation than the RES-ERα agonist complex, despite the fact that the percentages of helical residues are very similar in both the complexes.

**Figure 3:**
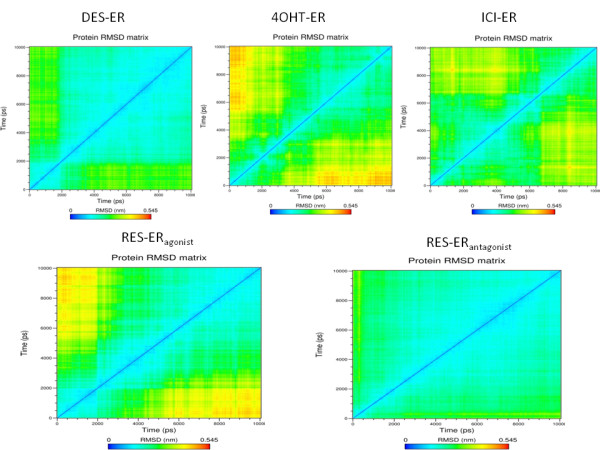
**RMSD matrices of ERα bound with different ligands computed from MD trajectory.** DES-ERα, RES-ERαantagonist and ICI-ERα display highly stable initial conformation of the protein. On the contrary, RES-ERαagonist complex shows similarity with 4OHT-ERα where the initial structure changes appreciably during the simulation. RMSD matrix has been computed using trajectory analysis tools available within GROMACS packages by comparing the root mean square distances of each structure in the trajectory with respect to each other structure and generate a bi-dimentional matrix.

To provide a detailed insight into all the visited conformations of ERα LBD during its MD simulation in the presence of various bound ligands, RMSD matrices have been computed, and the results are displayed in Figure 3. In the presence of pure agonist DES and antagonist ICI in the LBD, the structure of ERα is quite stable throughout the simulation period. It is to be noted that for DES-ERα complex, the MD simulation has been carried out starting from an agonist conformation in terms of Helix 12 position. Most of the conformations visited during the simulation are structurally very close to the initial agonist conformation; structurally distinct conformations are less frequently visited. The situation is in sharp contrast when 4-OHT, a SERM, is bound in the LBD. The starting conformation of ERα for the MD simulation is in antagonist conformation. Throughout the simulation period, this initial antagonist conformation is much less populated and structurally distinct conformations are more frequently visited. While for pure antagonist ICI, the starting structure of ERα for MD simulation is in antagonist conformation with respect to Helix 12 orientation and throughout the simulation period this antagonist conformation is highly populated and the structurally distinct conformations are very less frequently visited. This signifies that the binding of pure antagonist ICI, maintains the initial antagonist conformation better than a SERM. We now present the effect of RES on the stability of ERα conformation. When RES is bound in the agonist conformation of ERα LBD, the complex undergoes some structural changes during the initial simulation period and eventually stabilizes in the remaining simulation time with an RMSD of 0.35 nm from the initial structure. This feature is also evident when pure agonist DES is bound to the LBD. On the contrary, when RES is bound in antagonist ERα conformation, the initial antagonist structure is highly stable during the simulation and there is no structurally distinct conformational cluster visited during the simulation.

### Analysis of RES-ERα binding details and evaluation of binding energy

**Figure 4:**
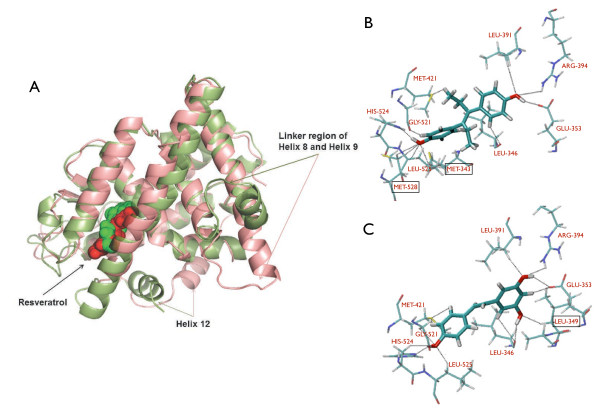
**Structures of RES-ERα complexes obtained from MD simulation. (A)** The structures of RES-ERαagonist (*green*) and RES-ERαantagonist (*red*) are overlapped. Using VMD, the ERα is shown in New Cartoon representation, and RES is shown in VDW mode. The side chains of some of the residues whose conformations are dramatically different between the complexes are shown: Helix12 and linker region of Helices 8 and 9. **(B)** &**(C)** Details of the hydrogen bonding contacts between DES and RES with ERα agonist conformation, respectively. RES is bound within the same ERα pocket that recognizes DES and 4OHT [30,32,33]. The hydrogen-bonding interactions with the different residues are shown.

We next analyzed the structure of RES bound ERα agonist and antagonist complexes (Figure 4). As evident from the figure, RES binds in the ligand binding site of ERα in a similar way for both the agonist and antagonist complexes. As mentioned earlier, the only difference in these two complexes are the Helix 12 orientation. In the agonist complex Helix 12 overlay over the ligand binding cavity while in the antagonist complex the Helix 12 orients in the co-activator binding groove. In the agonist RES-ERα complex, the N-terminal helical region of Helix 8 and the linker loop between Helices 8 and 9 moves upward with respect to the dimerization surface. We have recently demonstrated that the essential dimerization surfaces of ERα are mainly composed of Helix 10/11 and the C-terminal region of Helix 9 [34,35]. Thus the upward movement of the linker region between Helices 8 and 9 does not disrupt the essential dimerization surface of the receptor but may induce some perturbation therein.

Using the Linear interaction energy (LIE) method we calculate the binding free energies of RES with two different ERα conformations from the MD simulation average properties [36-40]. Lennard–Jones (LJ) and Coulomb interaction energies between the ligand and its environment were computed and averaged over the last 7 ns of the simulation. Subsequently, binding free energies (ΔG) were calculated by: ΔG = α (E^LJ^_BOUND_-E^LJ^_FREE_) + β (E^Coul^_BOUND_-E^coul^_FREE_) where E^LJ^ and E^Coul^ denotes the LJ interaction energy and the Coulomb interaction energy of the ligand when it is bound to the receptor (BOUND) and it is free in solution (FREE), respectively. The parameters α = 0.82 and β = 0.20 were used to calculate the binding energy which were developed for ERα-ligand systems and used frequently to calculate the binding free energy of different ligands to ERα [39,40].

In previous studies with cell based assays, DES is found to have stronger binding affinity to ERα compared to estradiol, but their binding energies are found to be quite comparable: -12.55 Kcal/mol for DES and -12.40 Kcal/mol for E2 [39]. For both DES and E2, the theoretical binding energies calculated from AMBER force-field for a given orientation are -10.43 Kcal/mol and -10.86 Kcal/mol, respectively [39]. Using the LIE method as adopted by Lipzig et al. [39] for ERα, but using the OPLS force-field, we found the binding energies of DES and RES are quite comparable in ERα agonist conformation: -9.5 for DES and -9.0 for RES. Interestingly, RES is found to bind strongly in the antagonist conformation of ERα with the calculated binding energy of -12.0 Kcal/mol compared to -9.0 Kcal/mol for ERα agonist conformation. Thus, in terms of binding energy, RES bound ERα antagonist complex is energetically more favorable than RES bound ERα agonist complex. This is an interesting revelation in the sense that the observed energetic preferences together with the orientational preference of Helix 12 in RES bound ERα complex provide possible pathways for antagonistic actions of RES.

Analyzing several structural parameters (RMSD, secondary structure, cluster analysis) and energetics, we can conclude that in RES bound ERα monomer, the Helix 12 preferentially orients to the co-activator binding grooves. This orientation is markedly different compared to the structure of ERα bound with pure agonist DES and rather similar with the conformation of ERα in presence of SERM 4-OHT.

In addition to the energetic preferences for binding, we further explored the details of the binding contacts of RES within ERα ligand binding cavity (Figure 4A, B). Figure 4A reveals that RES essentially occupies the same binding pocket in both the agonist and antagonist conformations of ERα which differ in the orientation of Helix 12. Although, as mentioned above, the orientation of Helix 12 in RES-ERα is similar with the Helix 12 orientation in 4-OHT-ERα complex, the RES binding pocket in ERα is very similar to that of DES. In fact, RES forms exactly similar hydrogen bonding contacts with eight residues as found in the case of DES binding with ERα: LEU 346, GLU 353, LEU 391, ARG 394, MET 421, GLY 521, HIS 524, and LEU 525 (Figure 4B & C). Interestingly, ER agonists like DES and E2 contain hydroxyl groups attached to the phenolic rings which seem crucial for agonistic action [32]. RES also contains similar hydroxyl groups (one additional) attached to phenolic rings which are involved in hydrogen bonding like in DES. Among them, hydrogen bonding with three key residues HIS 524, ARG 394 and GLU 353 are known to be crucial to elicit the agonistic effects of DES or E_2_ on ERα [32,33] and are also observed here in the complexes of RES bound ERα. Additionally, using alanine mutation and receptor binding studies with E_2_[41], it has been established that the residues GLY 521 and LEU 525 assume key role in recognizing the agonist ligand–both DES and RES are found to establish hydrogen bonding with these two key residues as well. Alanine scanning experiment revealed that while LEU 525 is crucial for ligand binding of any type regardless of its agonist or antagonist nature, HIS 524 is found to be important in the recognition of pure agonist ligands but not so for SERM like 4-Hydroxytamoxifen which lacks a second hydroxyl group [41]. RES possesses hydroxyl groups attached to two opposite benzene rings and the distal hydroxyl group is capable of interacting with HIS 524 through hydrogen bond. The similarity in the interaction pattern indicates that RES has the ability to elicit similar pharmacological effects (agonistic) on ERα like DES. However, there are explicit differences in the hydrogen bonding pattern as well: DES provides two additional hydrogen bonding with residues MET 343 and MET 528 while RES provides one additional hydrogen bonding with LEU 349 residue, as shown in Figure 4B & C within square. The interaction features of RES with ERα also shed light on the fact that RES has a greater affinity towards ERα compared to other phytoestrogens which exhibits higher affinity towards ERβ [42-44].

### Effect of RES binding on ERα-LBD dimers

**Figure 5:**
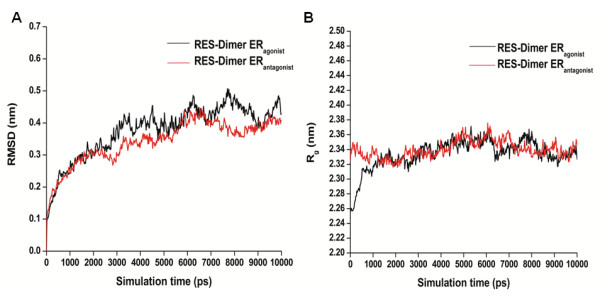
**Variations of RMSD (A) and radius of gyration, R**_**g **_**(B) of RES-ERα dimer complexes with simulation time.***Black* and *red* lines represent agonist and antagonist conformation of ERα, respectively.

Dimerization of ERα is essential for its transactivation functions [26,28]. We have analyzed the stability of RES binding on dimerized ERα in both agonist and antagonist forms (Figure 5). As seen in Figure 5A, during the first 3 ns of the simulation, both the systems undergo conformational readjustments according to the bound RES and monotonically reach an equilibrium state. Interestingly, RES bound ERα dimer in both agonist and antagonist dimer structures are stable during the dynamics. Molecular dynamic simulation reveals that the RES-ERα dimer antagonist complex is comparatively more stable than the agonist dimer complex. The average RMSD for the former dimer complex during the last 7 ns of simulation is 0.34 ± 0.06 nm while for the later dimer complex it has been observed to be 0.38 ± 0.08 nm with respect to the initial complex, respectively. We further analyze the stability of the dimer complex during the simulation in terms of the radius of gyration (Rg). R_g_ defines the overall shape and dimensions of the protein by calculating the mass-weighted root mean square distance of a collection of atoms from their common center of mass. The plot of the variation of radius of gyration of each LBD dimer with time is shown in Figure 5B. It is to be noted that we have considered two distinct conformations of ERα LBD dimer. Due to the orientational difference of Helix 12, the antagonist ERα dimer has a higher R_g_ compared to the agonist form. The R_g_ profile obtained from MD simulations reveals that the RES bound ERα dimer conformation where Helix 12 takes the classical antagonist orientation is more stable compared to the complex where Helix 12 takes the agonist conformation. In fact, the R_g_ values of the RES bound agonist dimer changes and converges with the R_g_ value of the antagonist ERα dimer during the simulation. Critical insight into the agonist dimer trajectory reveals that during the simulation period Helix 12 retains in its original agonist position. The observed changes in the R_g_ profile of the RES-ERα agonist dimer is solely attributed to the loop dynamics. Resveratrol bound ERα LBD dimer in agonist conformation is more stable compared to its monomeric form and the upward movement of the linker region between Helices 8 and 9, as observed for monomer, has not been observed for dimer. This can be explained by the fact that dimerization imposes constraints on the movement of the linker region, thus making the agonist dimer more stable compared to its monomeric form.

**Figure 6:**
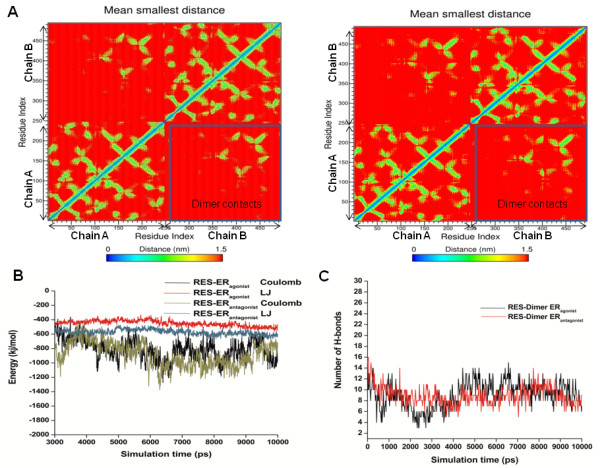
**Comparison of RES-ERα dimer characteristics. (A)***Left* and *right* figures represent RES-ERαagonist and ERαantagonist conformation of dimer, respectively. **(B)** Variations in Coulomb and LJ interaction energies between the two monomers of ERα bound with RES during the last 7 ns of simulation. *Black* and *red* colors represents Coulomb and LJ interaction between two monomer in agonist conformation, respectively, and *gray* and *cyan* colors represents Coulomb and LJ interaction between two monomers in antagonist conformation, respectively. **(C)** Variations in the number of hydrogen bonds between two monomers of ERα dimer bound with RES. *Black* and *red* line represents agonist and antagonist conformation of ERα, respectively.

Next we have analyzed the essential dimer contacts for RES bound agonist and antagonist dimer complexes, shown in Figure 6A and B, respectively. As evident from the contact map obtained from MD simulation, the regions of close contact residues between the two monomers are very similar for both RES bound agonist and antagonist ERα dimers. Further analysis reveals that in the case of antagonist dimer, the essential contacts between the two chains are more evident compared to those in the agonist dimer.

We further analyze the interaction energies and intermolecular hydrogen bonding between the two monomers of ERα dimer (Figure 6B, C). Resveratrol bound antagonist ERα forms more stable dimer than the agonist dimer (Figure 6B). Although the Coulomb interaction energies between the two monomers are very similar for both the agonist and antagonist conformations, van der Waals interactions are clearly more favorable in the antagonist dimer structure. In RES bound ERα antagonist dimer the average Coulomb and LJ interaction energies over the last 7 ns are -883.4 ± 167.7 kJ/mol and -572.2 ± 37.7 kJ/mol, respectively. In the case of RES bound ERα agonist dimer, the average coulomb and LJ interaction energies over the last 7 ns are -811.7 ± 147.3 kJ/mol and -449.7 ± 40.9 kJ/mol, respectively. Analysis of the hydrogen bonding contacts between the two monomers in the dimer interface reveals that both the complexes have very similar hydrogen bonding profile (Figure 6C). Throughout the simulation period, on average about 10-12 hydrogen bonds have been observed for both RES bound ERα agonist and antagonist dimers.

## Conclusions

In the present study, we have investigated ligand-specific responses of phytoestrogen RES on ERα LBD using molecular modeling and atomistic simulations. Our results shed light on the structural basis of the observed differential pharmacological effects of RES on ERα. We find that RES binds at the same ligand-binding pocket of ERα LBD that recognizes DES. To understand the structural effects of ligand-binding, we performed molecular dynamics simulation on ligand bound ERα LBD monomer and dimer complexes, and clarified the roles of parameters such as interaction energy, conformational energy changes, movement of the binding pocket residues, regions of high fluctuations (RMSF) and RMSD matrix of the protein, in conformational stability and plasticity of the receptor induced by the ligand.

When RES is bound to the agonist form of ERα, the dimer complex is more stable compared to its monomeric form and the upward movement of the linker region between Helix 8 and Helix 9, as observed for monomer, was not found for dimer. However, the binding of RES to ERα LBD monomer and dimer in antagonist conformation makes the complex more stable with higher binding energy than RES binding to ERα LBD agonist conformation. For ERα LBD agonist conformation, although RES binding interactions (binding pocket and hydrogen bonding interactions) is very similar to the binding feature of known agonist DES, the binding energy is much lower for RES than with DES and E2. This observation can explain partial estrogenecity of RES; to induce agonistic effects, potential co-activators have to displace the Helix 12 into an “agonist” conformation by directly competing with more preferable Helix 12 “antagonistic” orientation. Our results on RES–ERα complex are very similar to the genistein bound ERβ complexes where the relative free energies for the agonist and antagonist conformations of Helix-12 are similar with the “antagonist-like” state being slightly more stable [45]. In addition, in terms of Helix 12 orientation for agonist and antagonist conformations and the overall conformation plasticity during simulation, RES-ERα is markedly different from DES-ERα and rather shares characteristics with 4-OHT-ERα complex. It is known that the tissue selective agonism/antagonism of SERMs and phytoestrogens are the result of numerous factors, including structure of the ligand, over-expression of ER, availability of certain cellular proteins, balance of co-activators and co-repressors, to mention some [12,13,46-50]. This situation might occur when due to manipulations of endocrine therapy in breast cancer, estrogen levels, status of the receptor and cellular proteins are changes. Adjusting the balance between ligand-mediated structural perturbations of the ERα and tissue-specific cellular proteins will provide an adequate strategy for the use of RES in clinic.

## Methods

### Modeling of the receptor-resveratrol complex

The crystal structure of ERα LBD homo-dimer (PDB ID: 3ERD) where each monomer is bound with an agonist ligand DES was considered as ERα LBD agonist conformation. In this structure, Helix 12 is positioned over the ligand binding cavity such that co-activator binding pocket in the LBD remains unperturbed. On the other hand, the crystal structure of ERα LBD (PDB ID 3ERT) where a SERM, 4-hydroxytamoxifen (4-OHT), is bound to the LBD and the Helix 12 positioned itself in such a way that prevents co-activator binding, was considered for ERα antagonist conformation. Molecular docking using AutoDock 4.2 [51] was used to dock RES in both the agonist and antagonist conformation of ERα LBD. All the hetero atoms were deleted and non-polar hydrogens were merged for each receptor as required by AutoDock. The Kollman united-atom charge model was applied to the protein. Atomic solvation parameters and fragmental volumes were added to the protein. Resveratrol structure was obtained from the PUBCHEM chemical library (CID 445154). Rotatable bonds were assigned and non-polar hydrogens were merged for the ligand. For docking, the Partial atomic charges for the ligand were calculated using the Gasteiger-Marsili method.

Grid maps were generated by using the empirical free-energy scoring functions. A grid box of 120 × 120 × 120 grid points with a grid-point spacing of 0.375 Å was considered for docking. The box was centered such that it covered the entire LBD. 250 docking runs were performed and for each run, a maximum of 2,500,000 GA operations were carried out on a single population of 150 individuals. The default parameters of 0.8, 0.02 and 1 are used for crossover, mutation, and elitism weights, respectively. The lowest energy docked complexes of each ERα LBD monomer with RES was selected to build the dimer based on the ERα LBD dimer crystal structure (PDB ID: 3ERD) as a template. The modeled ERα LBD dimer complexed with ligand was then solvated, energy minimized, and appropriately relaxed with position restrained equilibration at 300 K to prepare for molecular dynamic simulations.

To prepare ERα LBD bound with pure antagonist ICI 182,780, (referred as ICI throughout the text), antagonist crystal structure 3ERT.pdb for the receptor has been used for molecular docking of ICI employing AutoDock 4.2 and a similar docking protocol as mentioned above.

### Molecular dynamics simulation

The parameters for the molecular dynamics simulation of RES, DES, 4-OHT and ICI were developed according to the OPLS force-field [52]. Each atom of RES molecule was assigned the proper atom type definition as per the OPLS-AA parameter set. The van der Waals and torsional parameters and the atomic partial charges for the ligand were obtained by group analogy in the OPLS-AA set. The atomic partial charges are readjusted to maintain the charge neutrality of the whole molecule. The parameters are tested by comparing the GROMACS [53,54] energy minimized structures with the energy minimized structures obtained from plane wave based DFT calculations using CPMD [55].

Each ERα LBD monomer and the dimer complex were subjected to a preliminary short energy minimization in vacuo using the steepest descent algorithm. Then the system was solvated with SPC explicit water model in a cubic box with periodic boundary condition. The box dimension was chosen such that all the protein atoms were at a distance equal to or greater than 1 nm from the box edges. The ionization state of the residues were set to be consistent with neutral pH and Na^+^ ions were added to make the system charge-neutral. The solvated system was then subjected to a second energy minimization with 500 steps of steepest descent algorithm to eliminate any bad contacts with water. After that, a 500 ps position restrained dynamics was carried out where the complex was restrained by restraining forces while the water molecules were allowed to move freely. It was then followed by 200 ps of NVT simulation at 300 K and 200 ps of NPT simulation to achieve proper equilibration of the system to be simulated. Final production simulations were performed in the isothermal-isobaric (NPT) ensemble at 300 K, using an external bath with a coupling constant of 0.1 ps. The pressure was kept constant (1 bar) by using pressure coupling with the time-constant set to 1 ps. The LINCS [56] algorithm was used to constrain the bond lengths involving hydrogen atoms, allowing the use of 2.0 fs time step. The Van der Waals and Coulomb interactions were truncated at 1.4 nm and the SHIFT algorithm as implemented in GROMACS has been used to minimize the error from truncation. The trajectories were stored at every 5 ps.

Structural analysis were carried out by using the in-built tools of GROMACS and the secondary structure assignments were carried out with DSSP [57] module integrated with GROMACS. The RMSD matrices were computed on each of the trajectories by the least square fitting of main-chain atoms and the matrices were then processed to extract clusters of similar conformations.

## Competing interests

The authors declare that they have no competing interests.

## Authors’ contributions

SC participated in the planning of the study, carried out all the simulations, organized the data, analyzed and interpreted the results, prepared the figures and tables, and helped in drafting the manuscript. ASL brought the problem of Resveratrol action on Estrogen Receptors into attention, analyzed and interpreted the results and helped drafting the manuscript. PKB planned the study with ASL and SC, analyzed and interpreted the results, and drafted and finalized the manuscript. All the authors have read and approved the final manuscript.

## Authors’ information

Pradip K Biswas: Website: https://www.tougaloo.edu/pbiswas.
